# Frequency of benign neutropenia among Black versus White individuals undergoing a bone marrow assessment

**DOI:** 10.1111/jcmm.17346

**Published:** 2022-06-01

**Authors:** Scott C. Borinstein, David Agamasu, Jonathan S. Schildcrout, Lisa Bastarache, Minoo Bagheri, Lea K. Davis, Dan M. Roden, C. Michael Stein, Sara L. Van Driest, Jonathan D. Mosley

**Affiliations:** ^1^ 12328 Department of Pediatrics Vanderbilt University Medical Center Nashville Tennessee USA; ^2^ 5708 Meharry Medical College Nashville Tennessee USA; ^3^ 12328 Department of Biostatistics Vanderbilt University Medical Center Nashville Tennessee USA; ^4^ 12328 Department of Biomedical Informatics Vanderbilt University Medical Center Nashville Tennessee USA; ^5^ 12328 Department of Medicine Vanderbilt University Medical Center Nashville Tennessee USA; ^6^ 12328 Department of Pharmacology Vanderbilt University Medical Center Nashville Tennessee USA

**Keywords:** bone marrow biopsy, neutropenia, racial differences

## Abstract

Healthy individuals in the United States identified as having Black race have lower neutrophil counts, on average, than individuals identified as having White race, which could result in more negative diagnostic evaluations for neutropenia. To test this hypothesis, the proportion of evaluations where the final diagnosis was clinically insignificant neutropenia for Black and White individuals who underwent an evaluation by a haematologist that included a bone marrow (BM) biopsy to investigate neutropenia was assessed. 172 individuals without prior haematological diagnoses who underwent a haematological evaluation to investigate neutropenia. Individuals diagnosed with clinically insignificant neutropenia between Black and White individuals were compared using a propensity‐score‐adjusted logistic regression. Of 172 individuals, 42 (24%) were classified as Black race, 86 (50%) were males, and the 79 (46%) were over 18 years old. A BM biopsy did not identify pathology in 95% (40 of 42) of Black individuals and 68% (89 of 130) of White Individuals. Black individuals (25 of 42 [60%]) received a final diagnosis of clinically insignificant neutropenia, compared to White individuals (12 of 130 [9%]) (adjusted odds ratio =7.9, 95% CI: 3.1 – 21.1). We conclude that black individuals were more likely to receive a diagnosis of clinically insignificant neutropenia after haematological assessment.

## 1 INTRODUCTION

Population studies in the United States have found that, on average, populations identified as Black race have lower neutrophil counts compared to populations of White race, a phenomenon sometimes referred to ‘‘Benign Ethnic Neutropenia’.^1^, ^2^, ^3^, ^4^ This difference is largely attributable to homozygosity for the benign genetic variant rs2814778‐C in the promoter of the Atypical Chemokine Receptor 1 (ACKR1) gene.^5^, ^6^ Up to 65% of African–Americans in the United States are homozygous for the rs2814778‐C variant (the Duffy Null phenotype).^7^, ^8^, ^9^, ^10^ In contrast, homozygosity for this variant is rare among White individuals. Because this neutrophil‐lowering genotype is strongly confounded with race, clinical decision‐making related to the evaluation of neutropenia could differentially impact racial groups.^11^

Lower baseline neutrophil counts could predispose Black individuals to undergo clinical evaluations, including invasive diagnostic procedures such as bone marrow biopsy (BMB), to identify a pathological cause contributing to the lower counts. Since the lower neutrophil counts among rs2814778‐CC genotype carriers are not associated with underlying disease or other clinically meaningful sequelae, these investigations would not be expected to identify underlying pathology.^12^ We hypothesized that, because of the high frequency of the rs2814778‐CC genotype among individuals of Black race, this predisposition to lower neutrophils could result in a measurable race‐related healthcare disparity. To test this hypothesis, we analysed a clinical data set to determine whether Black individuals who underwent an evaluation by a haematologist that included a BMB to investigate a finding of neutropenia were more likely than White individuals to be diagnosed with a clinically insignificant neutropenia.

## 2 METHODS

### 2.1 Study population

Individuals were identified using Vanderbilt University Medical Center's (VUMC) Synthetic Derivative (SD) resource, which is a deidentified data set comprising a large portion of data captured through the medical centre's electronic health record (EHR).^13^ The SD database currently contains records for over 2.8 million individuals, with no defined exclusions. The study population was derived from a set of 20,353 individuals with a race recorded as either ‘White’ or ‘Black’ in the EHR, and a BMB pathology report in their clinical records. Individuals with an established diagnosis based on ICD‐9/ICD‐10 codes for a haematological cancer, a blood transfusion, an organ transplant or chemotherapy/radiation therapy appearing 3 or more days prior to their first biopsy were excluded. A keyword search was then used to identify all BMB reports that contained any of the phrases (‘leukopenia’, ‘leucopenia’, ‘low white’, ‘decreased white’, ‘reduced white’, ‘low wbc’, ‘decreased wbc’, ‘reduced wbc’, ‘neutropenia’, ‘low neutro’, ‘decreased neutro’, ‘reduced neutro’, ‘lymphopenia’, ‘ anc’, ‘anc’, ‘lymphopenia’, ‘granulopenia’ and ‘agranulocytosis’) as part of the clinical indication for the biopsy. The clinical record of each individual was manually reviewed. As part of the manual review, only those individuals with a BMB performed at VUMC that included an indication of low neutrophils listed in their BMB report and a clinical evaluation by a haematologist were retained. Those with a prior diagnosis of haematological disease, a prior BMB or previously diagnosed cancer identified by either automated screening using ICD‐9/ICD‐10 codes or manual review were excluded.

This study was evaluated by the VUMC Institutional Review Board and determined to be non‐human subjects research.^13^

### 2.2 Clinical data

Race, gender, as recorded in the EHR, and Duffy antigen testing results were extracted from structured data tables. Age was based on the date of the first BMB report. White blood cell count, absolute neutrophil count (ANC), platelet count and haemoglobin levels were extracted from the haematology clinical note preceding the BMB. All haematology notes prior the BMB were reviewed and findings of concern to the haematologist (in addition to neutropenia) were coded including haematological abnormalities (anaemia, thrombocytopenia, cell type elevations, presence of blast cells, thrombosis, gamma globulin disorders and other), comorbidities (rheumatological disease, splenomegaly, trisomy 21 and other) and symptoms (fever, concerns for recurrent infections, bone pain, lymphadenopathy, night sweats and unintentional weight loss, and other). Presence or absence of benign neutropenia in the stated differential diagnosis was recorded. For each individual, the first BMB hematopathology report, which comprised histology, flow cytometry, immunochemistry and/or other relevant studies was reviewed. All haematology notes after the BMB were reviewed to identify the final diagnosis explaining the individual's neutropenia. An individual was assigned a diagnosis of ‘clinically insignificant neutropenia’ if the final diagnosis was ‘benign [ethnic] neutropenia’ or neutropenia of no clear aetiology and assessed to likely be of no clinical significance with respect to adverse health outcomes. If the final assessment included several possible diagnoses, the diagnoses was coded as ‘undetermined’; and if the evaluation was not completed because the patient did not return for follow‐up, the diagnosis was ‘lost to follow‐up’. All clinical data were reviewed by a physician blinded to the individual's race (JDM). Each hematopathology assessment was then categorized as either having a clinically significant haematological or cytogenetic abnormality, such as evidence of malignancy or myelodysplastic syndrome, myelofibrosis, maturation arrest, significant hypoplasia suggestive of aplastic anaemia, etc. as defined by a haematologist (SCB). Upon review and interpretation of the report, each BMB was categorized as normal or abnormal. Data were extracted to a REDCap database.^14^, ^15^ Bone marrow biopsies were performed that typically also include analysis of the aspirate as well as the decalcified biopsy.

### 2.3 Analysis

Frequencies and median values were computed for the overall sample and stratified by race (Black vs. White) and stratified by whether the final diagnosis was ‘clinically insignificant neutropenia’ versus other diagnoses. Differences in ANC levels between Black and White individuals were assessed by a Mann–Whitney U test. Unadjusted associations of a clinically insignificant neutropenia diagnosis with age, gender and other baseline variables were studied with separate logistic regression analyses using biopsy outcome as the dependent variable and the baseline characteristic as the independent variable. The p‐value for the independent variable is reported.

The proportion of individuals with a diagnosis of clinically insignificant neutropenia was calculated separately for Black and White individuals and is presented for the overall study sample and stratified by select baseline characteristics. To test the independent association between clinically insignificant neutropenia and Black race, as compared to White race, multivariable logistic regression was used where the diagnosis of clinically insignificant neutropenia was the dependent variable and Black race as the independent variable. To control for factors associated with being of Black race, the regression model included a propensity‐score for being in the Black race group. Weights for the propensity‐score components were determined by logistic regression analysis using Black race as the dependent variable and the following independent variables: age, age‐squared, ANC, ANC‐squared, haemoglobin levels, platelet count, a clinical diagnosis of anaemia, a clinical diagnosis of thrombocytopenia, presence of any haematological abnormality, presence of any symptom, presence of any other comorbidity, evaluation in inpatient setting, neutropenia associated with acute illness. The propensity‐score for each individual was the predicted probability of belonging to the Black race group. The final model reported here excludes 2 participants (one Black and one White participant) with outlying propensity‐score weights. The results when these individuals were included differed modestly and is presented in the referenced supplementary tables.

All analyses used 2‐sided tests and, unless otherwise noted, a *p*‐value < 0.05 was considered significant.

## 3 RESULTS

### 3.1 Baseline characteristics

**FIGURE 1:**
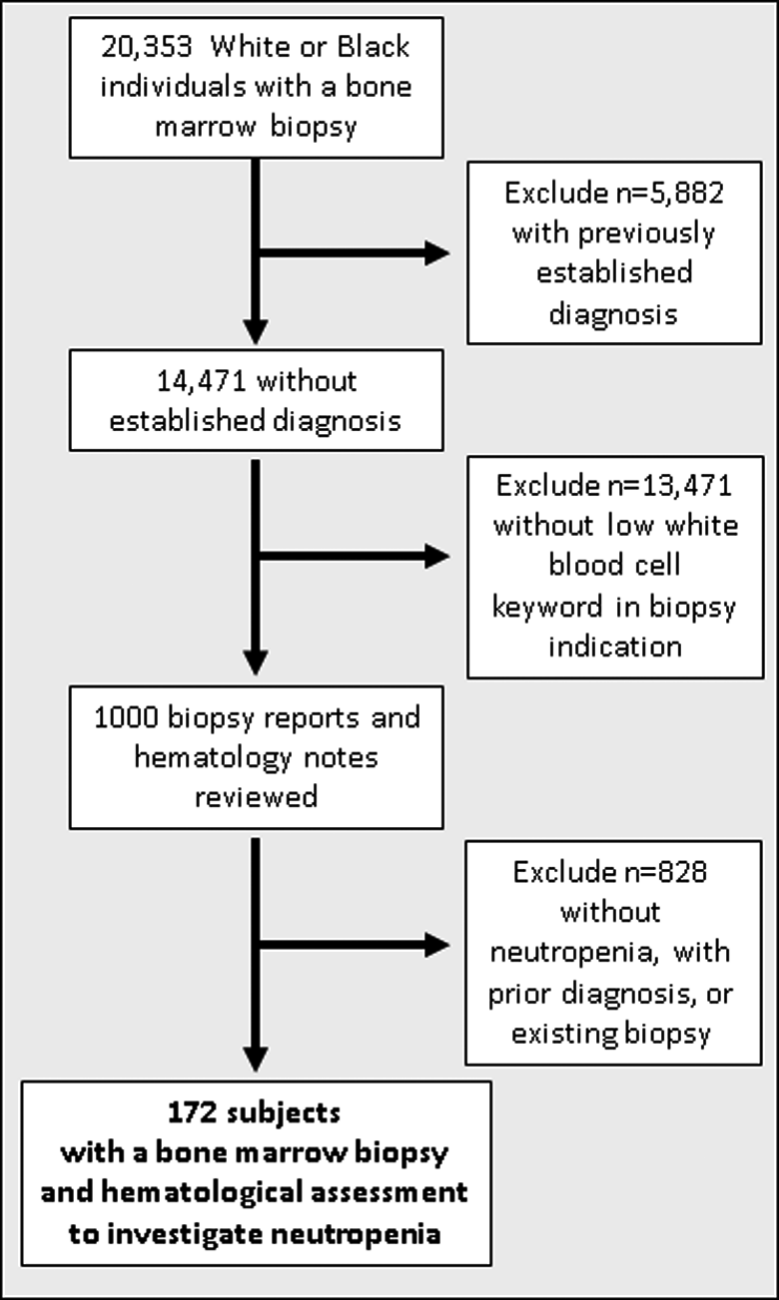
Selection of the bone marrow biopsy population. The study population was drawn from individuals identified through a Vanderbilt University Medical Center de‐identified electronic health record resource

**FIGURE 2:**
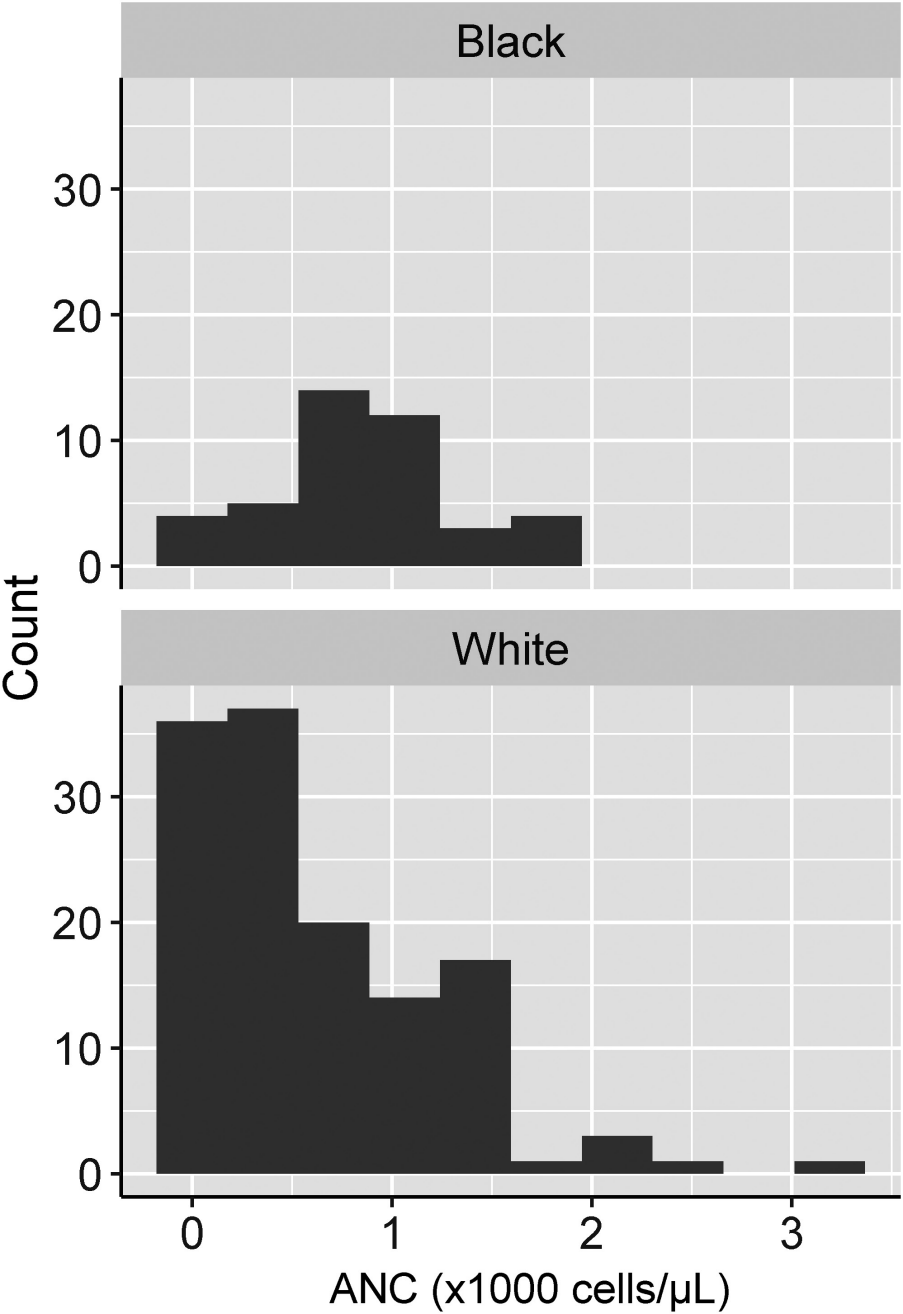
Frequency histograms showing the distribution of absolute neutrophil count (ANC), by race

After exclusions, there were 172 individuals who had a haematological evaluation including a BMB for a clinical indication that included neutropenia and without a previously established haematological diagnosis (Figure 1). Among these, 42 (24%) were classified as Black race and 86 (50%) were males (Table 1). The median age was 14 years and 79 (46%) of subjects were 18 years or older with a higher median age of Black individuals (22.4 years [interquartile range, 5.3 – 36.1]) than White individuals (13.1 [IQR, 2.0 – 50.6]) (Figure S1). The median absolute neutrophil (ANC) count was significantly higher among Black individuals (830 cells/µl [IQR, 667 – 1000]) than among White individuals (460 [IQR, 145 – 985]) (*p *= 0.002, Mann–Whitney U test), and 21% versus 53.1%, respectively, had an ANC <500 cells/µl (Figure 2).

**TABLE 1:** Characteristics of the study population by race group

Characteristic^1^	All (*n* = 172)	Black (*n* = 42)	White (*n* = 130)
Males	86 (50%)	23 (54.8%)	63 (48.5%)
Age (years)	14.4 (2.5 – 45.4)	22.4 (5.3 – 36.1)	13.1 (2.0 – 50.6)
WBC count (×1000 cells/µl)	3.2 (2.1 – 4.4)	3.2 (2.5 – 4.2)	3.2 (1.9 – 4.5)
ANC (×1000 cells/µl)	0.6 (0.2 – 1.0)	0.8 (0.7 – 1.0)	0.5 (0.1 – 1.0)
Platelet count (×1000/µl)	212 (148 – 285)	225 (188 – 274)	210 (144 – 304)
Haemoglobin (g/dl)	11.9 (10.1 – 13.1)	12.2 (11.2 – 13.4)	11.7 (9.9 – 13.1)
BM biopsy performed in outpatient setting	130 (75.6%)	37 (88.1%)	93 (71.5%)
Neutropenia associated with acute illness	57 (33.1%)	9 (21.4%)	48 (36.9%)
Clinically insignificant neutropenia on differential diagnosis	30 (17.4%)	19 (45.2%)	11 (8.5%)
Haematologist concerns^2^			
Anaemia	45 (26.2%)	7 (16.7%)	38 (29.2%)
Thrombocytopenia	35 (20.3%)	5 (11.9%)	30 (23.1%)
Elevated cell count	4 (2.3%)	0 (0%)	4 (3.1%)
Concern for blast cells	5 (2.9%)	0 (0%)	5 (3.8%)
Gamma globulin disorder	3 (1.7%)	0 (0%)	3 (2.3%)
History of thrombosis	2 (1.2%)	1 (2.4%)	1 (0.8%)
Other haematological problem	11 (6.4%)	1 (2.4%)	10 (7.7%)
Fever	36 (20.9%)	7 (16.7%)	29 (22.3%)
Immune dysfunction	16 (9.3%)	1 (2.4%)	15 (11.5%)
Night sweats, weight loss	9 (5.2%)	3 (7.1%)	6 (4.6%)
Bone pain	5 (2.9%)	1 (2.4%)	4 (3.1%)
Lymphadenopathy	3 (1.7%)	0 (0%)	3 (2.3%)
Other symptoms	14 (8.1%)	0 (0%)	14 (10.8%)
Rheumatologic disease	19 (11%)	3 (7.1%)	16 (12.3%)
Splenomegaly	2 (1.2%)	1 (2.4%)	1 (0.8%)
Trisomy 21	2 (1.2%)	0 (0%)	2 (1.5%)
Other comorbidities	10 (5.8%)	2 (4.8%)	8 (6.2%)

^1^ Continuous variables show median (interquartile range) and categorical variables shown (%).

^2^ Other relevant diagnoses identified by the haematologist.

Overall, 49 (29%) individuals had no comorbidities or symptoms other than neutropenia (isolated neutropenia) that were of concern to the haematologist. The proportions of isolated neutropenia among were 41% for Black and 25% for White individuals. The respective proportions of Black individuals with any concerning haematological abnormalities, comorbidities, or symptoms were 31%, 14% and 29%, and for White individuals were 49%, 19% and 41% (Table 1). Black individuals were more likely to have clinically insignificant neutropenia included among the differential diagnoses, as compared to White individuals (45.2% vs. 8.5%).

### 3.2 Bone marrow evaluations

Black individuals were more likely than White individuals to have a bone marrow biopsy (often including flow cytometry and/or cytogenetic evaluation) that did not identify an abnormality. Forty of 42 (95%) of Black individuals versus 89 of 130 (68%) of White individuals, had a normal biopsy. The proportions with a normal biopsy were similar among individuals with isolated neutropenia by racial groups (94% [16 of 17] for Black individuals versus 91% [29 of 32] for White individuals). Among individuals with neutropenia and another haematological abnormality, comorbidity, or symptom, the normal biopsy proportion was higher for Black individuals (96% [24 of 25], as compared to White individuals (61% [60 of 98]) (Table S1).

### 3.3 Diagnosis by haematologist

Overall, 37 (22%) individuals were diagnosed with a clinically insignificant neutropenia. Baseline characteristics significantly associated with an increased likelihood of a clinically insignificant neutropenia included older age, higher ANC, higher haemoglobin, absence of a fever, and having isolated neutropenia (Table S2).

**FIGURE 3:**
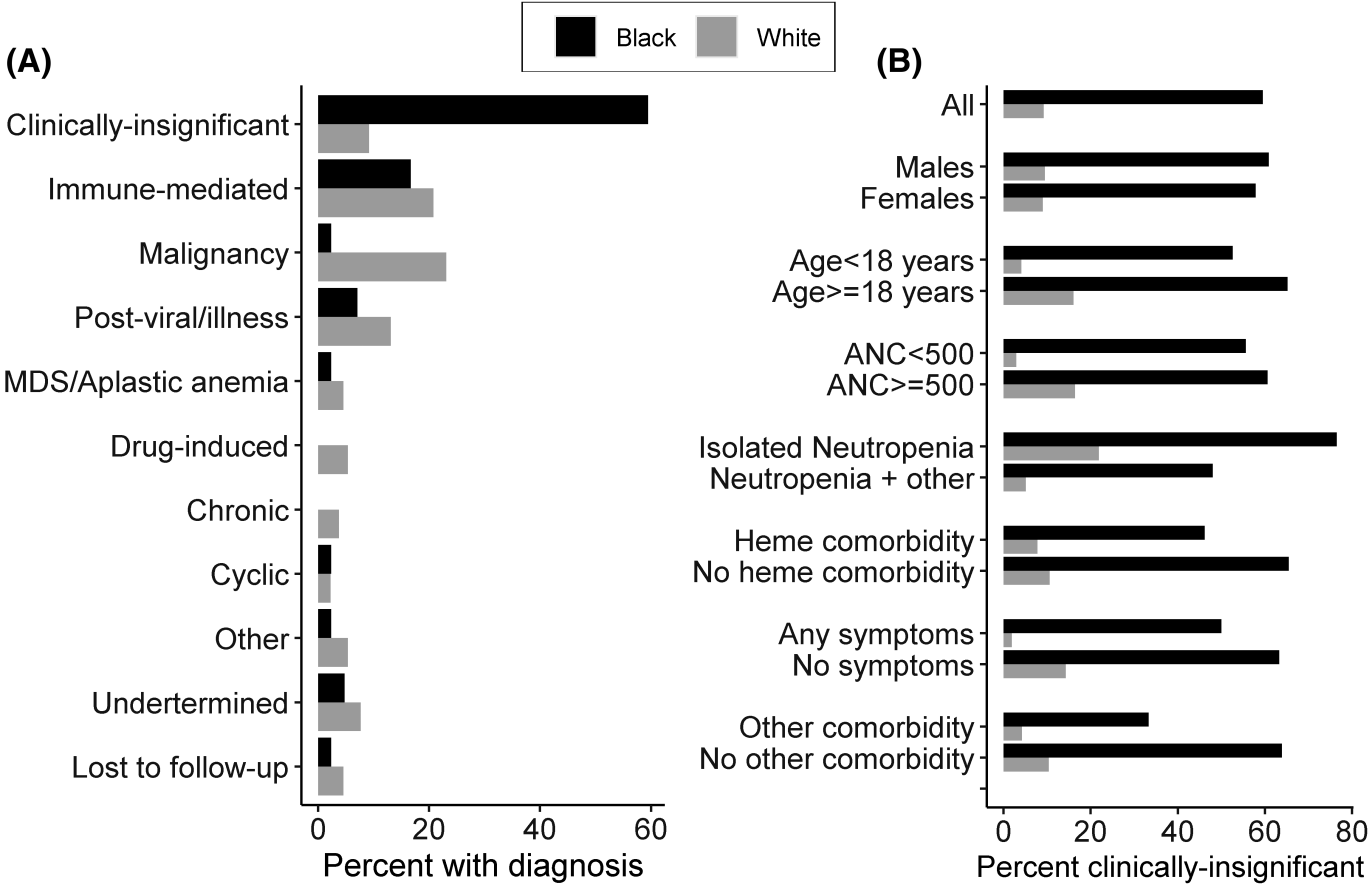
Diagnoses of the causes of low neutrophil counts made by the haematologist for Black and White individuals. (A) Frequency histogram showing the proportion of Black and White participants with a given diagnosis explaining their neutropenia. The subgroup: ‘Other’ includes (congenital neutropenia, nutritional deficiency, hypersplenism, WHIM syndrome, CVID, hypogammaglobulinaemia, myelofibrosis, hepatitis C). (B) Proportion of black and white individuals with a diagnosis of clinically insignificant neutropenia, stratified by select population characteristics

The majority of Black individuals (25 of 42 [60%]) were diagnosed with clinically insignificant neutropenia (Figure 3A). In contrast, 12 of 130 (9%) of White individuals were given this diagnosis. This large difference in proportions by race persisted when the data were stratified by isolated neutropenia, gender, age, ANC (>500 vs. ≤500 cell/µl) and the presence of comorbidities or symptoms (Figure 3B). Among Black individuals, the proportions with clinically insignificant neutropenia were similar among individuals with ANC >500 (56%) and ANC ≤500 cell/µl (61%). Only 1 Black individual underwent Duffy antigen testing and this individual had the Duffy Null phenotype and a final diagnosis of clinically insignificant neutropenia.

In a multivariable logistic regression model adjusted for a propensity‐score for factors associated with Black versus White race, Black race was associated with a significantly increased probability of having a final diagnosis of clinically insignificant neutropenia (odds ratio =7.9, 95% CI: 3.1 – 21.1, *p* = 9 × 10^−7^) (Table S3).

## 4 DISCUSSION

Approximately 60%–65% of African–Americans carry the Duffy Null phenotype that associates with lower neutrophil counts. The rs2814778‐C single nucleotide polymorphism underlying this genotype confers the Duffy Null red blood cell antigen phenotype [Fy(a‐b‐)].^16^, ^17^, ^18^ The lower ANC associated with the Duffy Null phenotype is sometimes referred to as Benign Ethnic Neutropenia and is clinically insignificant, as individuals have normally functioning bone marrow and immune systems and do not have an increased incidence of infections.^19^, ^20^ Their average ANC may lie between 500–1,500, below the commonly accepted threshold for neutropenia (ANC <1500 cells/µl).^21^

We observed that Black individuals with a clinical history highly suggestive of a clinically insignificant neutropenia due to the Duffy Null phenotype, including an ANC >500 and an absence of clinical comorbidities, underwent an extensive clinical work‐up for neutropenia that included a bone marrow biopsy. The distributions of ANC values by race highlight that individuals of Black race were more frequently biopsied for modest neutropenia (ANC >500) than individuals of White race. These data suggest that Black individuals are more susceptible to an unnecessary, invasive, and painful procedure than White individuals due to frequent carriage of a benign genotype. Furthermore, the cost of a bone marrow biopsy at our medical centre is approximately $6000, significantly less than Duffy antigen testing, which costs less than $200 when performed in most laboratories and blood banks.^12^ Omission of an unnecessary BMB would decrease health care utilization costs. We suggest that an important contributor to an unnecessary diagnostic odyssey among Black individuals is that established normal references ranges for ANC values are based on a predominantly White (with a predominately Duffy positive phenotype) population. Furthermore, this study demonstrates that these unsuitable reference ranges contribute to a measurable health disparity that disproportionately impacts Black individuals.

Many clinicians are aware of an increased frequency of clinically insignificant neutropenia among Black individuals. Consistently, clinically insignificant neutropenia was often included among the differential diagnosis for neutropenia among Blacks in this study. When neutropenia is observed, current clinical guidelines for identifying clinically insignificant neutropenia include serial monitoring of ANC (at least 2 values usually 3–6 months apart) along with careful physical exam and history. In some instances, a complete blood count in conjunction with clinical factors (family history, infection history, history of oral infections or lesions) can implicate congenital neutropenias as the aetiology.^22^ If ANC values remain stable, and in the absence of concerning symptoms (i.e., an isolated neutropenia), a bone marrow biopsy evaluation is typically not warranted.

We would advocate that assessing for the Duffy Null genotype (by genetic testing) or phenotype (by antigen detection) also be a standard part of the work‐up for a WBC or ANC value that is concerning to a provider, prior to more extensive evaluation or serial monitoring, and regardless of race.^16^ Knowing whether an individual carried the Duffy Null phenotype would indicate whether a lower ANC value may reflect a benign predisposition. In support of a role for genotyping/phenotyping, a retrospective study of individuals of Black race treated at three medical centres who had a BMB to investigate an isolated low white blood cell count, 97% of individuals carried the rs2814778‐CC (Duffy Null) genotype.^12^ Furthermore, 97% of the biopsies among genotype carriers demonstrated benign findings. In the absence of knowing the Duffy phenotype status, there is uncertainty as to whether an observed low ANC value is expected for an individual, and this uncertainty may contribute to an extensive haematological work‐up.

Often the Duffy phenotype is not measured clinically, but inferred based on an individual's race and observed ANC. Consistently, in this study, only 1 Black individual had documented antigen testing, indicating that, even among haematologists, testing may not be a routine part of a clinical work‐up. However, an inference that an individual carries the Duffy Null phenotype could be inaccurate over 40% of the time among African–Americans. Furthermore, incorrectly assuming a Black individual has the Duffy Null phenotype could lead a provider to conclude that a low ANC is of no clinical significance, possibly resulting in a delayed diagnosis.

We suspect that many of the Black patients in this study received an intensive haematological work‐up, including a BMB, because their neutrophil counts fell below an established clinical reference range that is not calibrated to their underlying genetic predisposition. While an ANC<1500 cells/µl is the commonly accepted threshold for neutropenia, a recent study observed that up to 5% of Black individuals with the Duffy Null phenotype may have a value that falls below this threshold.^23^ In contrast, less than 0.1% of Black individuals without the null phenotype fell below this value. Thus, Black individuals with this benign phenotype are considerably more likely to have a value that is considered to be clinically abnormal and are at increased risk of an unnecessary clinical work‐up. In this study, 65% of Black individuals with an ANC <500 cells/µl were diagnosed with clinically insignificant neutropenia, suggesting that the phenotype may predispose to considerably lower ANC values. Separate clinical reference ranges for those with and without the Duffy Null phenotype would address this disparity.

We examined a clinical endpoint that included an evaluation by a haematologist in order to ensure that the final diagnosis of clinically insignificant neutropenia was accurate. However, the underlying problem of health care disparities among those with the Duffy Null phenotype manifests in other, and sometimes more subtle, contexts beyond repeated testing for low WBC levels. For instance, these individuals are more likely to have medications stopped or delayed because of a low ANC not recognized to be related to the Duffy Null phenotype.^24^, ^25^, ^26^ They are also more likely to be excluded from clinical trials and other studies that have ANC thresholds for inclusion.^27^, ^28^ Again, many of these disparities could be attenuated with WBC and ANC reference ranges calibrated to the underlying biology.

It is important to note that, in the absence of isolated neutropenia, the clinical decision‐making process surrounding neutropenia becomes more complex. Thus, when Black individuals with clinically insignificant neutropenia due to the Duffy Null phenotype present with additional symptoms such as anaemia, thrombocytopenia, fever, lymphadenopathy, weight loss, or fatigue, their low ANC may influence medical decision‐making and these patients may undergo a more extensive diagnostic work‐up to rule out more serious medical conditions. Abnormalities in two cell lines can be suggestive of an underlying bone marrow disorder. However, if the lower value for neutrophils is due to a benign predisposition, the likelihood of an underlying bone marrow predisposition is lower. Consistently, in this study, individuals of Black race, as compared to those of White race, were less likely to have an underlying bone marrow abnormality, even with a second haematological abnormality. Thus, their predisposition to a lower ANC is likely contributing to incorrect clinical inferences. Further studies would be needed to ascertain whether genotype‐specific references ranges would avoid unnecessary procedures.

The age distributions differed between the races. For White individuals, 37% were under 10 years old and 26% were over 50 years old. In contrast, for Black individuals, the respective percentages were 24% and 9%. Haematological malignancies are more common in early or late life, so the bimodal age distribution among White individuals corresponds to this expected disease pattern. In contrast, Black individuals were more likely to undergo evaluations in older childhood to middle life when leukaemia or other bone marrow diseases occur less frequently. Such a pattern would be expected if the haematological abnormality (neutropenia) of clinical concern was not driven by underlying disease.

This study has limitations. The data came from a single tertiary care medical centre and the study population and results may not be generalizable to other communities. Racial groups are comprised of heterogeneous collections of individuals representing a range of ancestries; binning individuals into two groups largely based on skin colour ignores important biological differences.^29^ In addition, race assignment in the EHR may represent either self‐reported race or race assigned by others. Data were derived from EHRs and reflect only the care obtained within this care system and do not include encounters from outside facilities. The bone marrow and final diagnoses represent the assessments of the pathologists and haematologists and the complete set of data used in these assessments were not available for re‐review in this de‐identified data resource. Black and White individuals differed with respect to several relevant baseline characteristics (age, ANC count, burden of comorbidities), which could reflect a different predisposition toward neutropenia or selection biases related to race and referral for evaluation.

In summary, this retrospective study found that Black individuals undergoing an evaluation by a haematologist that included a BM biopsy for neutropenia were more likely to be diagnosed with a clinically insignificant neutropenia of no clinical significance, as compared to White individuals. We speculate that this is due to the benign Duffy Null phenotype/genotype that is common among Black individuals and that is associated with lower neutrophil counts. Establishing reference ranges based on the underlying this phenotype/genotype may avoid healthcare disparities attributable to the lower ANC counts in this group.

## CONFLICT OF INTEREST

SCB serves as a consultant for Foundation Medicine. No other authors have a conflict of interest.

## AUTHOR CONTRIBUTIONS

**Scott C. Borinstein:** Conceptualization (equal); Data curation (equal); Formal analysis (equal); Investigation (equal); Methodology (equal); Project administration (equal); Writing – original draft (equal); Writing – review & editing (equal). **David Agamasu:** Formal analysis (equal); Investigation (equal); Writing – original draft (equal); Writing – review & editing (equal). **Jonathan S Schildcrout:** Data curation (equal); Formal analysis (equal); Investigation (equal); Writing – original draft (equal); Writing – review & editing (equal). **Lisa Bastarache:** Conceptualization (equal); Data curation (equal); Formal analysis (equal); Investigation (equal); Writing – original draft (equal); Writing – review & editing (equal). **Minoo Bagheri:** Formal analysis (equal); Investigation (equal); Methodology (equal); Writing – original draft (equal); Writing – review & editing (equal). **Lea K Davis:** Formal analysis (equal); Investigation (equal); Methodology (equal). **Dan M Roden:** Conceptualization (equal); Data curation (equal); Formal analysis (equal); Investigation (equal); Methodology (equal); Writing – original draft (equal); Writing – review & editing (equal). **Michael Stein:** Conceptualization (equal); Data curation (equal); Formal analysis (equal); Investigation (equal); Methodology (equal); Writing – original draft (equal); Writing – review & editing (equal). **Sara L Van Driest:** Conceptualization (equal); Data curation (equal); Formal analysis (equal); Investigation (equal); Methodology (equal); Writing – original draft (equal); Writing – review & editing (equal). **Jonathan D Mosley:** Conceptualization (equal); Data curation (equal); Formal analysis (equal); Funding acquisition (equal); Investigation (equal); Methodology (equal); Writing – original draft (equal); Writing – review & editing (equal).

## IRB APPROVAL

This study was evaluated by the Vanderbilt University Medical Center (VUMC) Institutional Review Board prior to data collection and determined to be non‐human subject's research.

## Supporting information

Supplementary Material

## Data Availability

Access to data from the VUMC resources can be requested through the investigators, and requires completing appropriate data use and sharing agreements and IRB approvals.
