# Supplementary material for: Frequency of benign neutropenia among Black versus White individuals undergoing a bone marrow assessment
Source: J Cell Mol Med. 2022 Jun 1;26(13):3628–35. doi: 10.1111/jcmm.17346 (PMC9258701; doi:10.1111/jcmm.17346)

**SUPPLEMENTARY Figures**

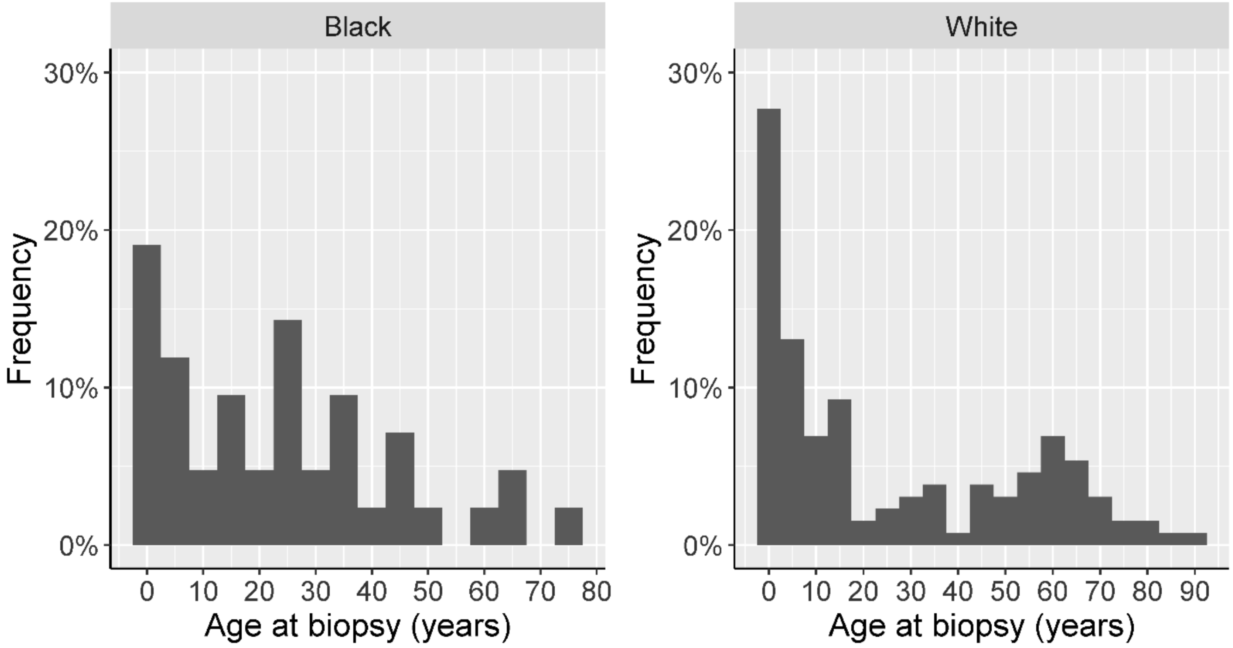


**Supplementary Figure 1:** Age distributions of age at biopsy for Black and White subjects.

## SUPPLEMENTARY TABLES

### Supplementary table 1: Population characteristics, stratified by results of bone marrow biopsy analysis.

| Variable | Normal biopsy (n=129) | Abnormal biopsy (n=42) |
|---|---|---|
| Black race | 40 (31%) | 2 (4.7%) |
| Males | 68 (52.7%) | 18 (41.9%) |
| Age <18 years old | 75 (58.1%) | 18 (41.9%) |
| WBC count (x1000/mm3) [Median (IQR)] | 3.2 (2.3 - 4.5) | 2.9 (2.0 - 4.0) |
| ANC (x1000/mm3) [Median (IQR)] | 0.73 (0.28 - 1.23) | 0.52 (0.25 - 1.10) |
| Platelet count (x1000/mm3) [Median (IQR)] | 223 (167 - 308) | 176 (115 - 226) |
| Hemoglobin (g/dl) [Median (IQR)] | 12.2 (10.8 - 13.4) | 10.3 (8.6 - 12.0) |
| ANC <500 cells/mm3 | 55 (42.6%) | 23 (53.5%) |
| Neutropenia of no clinical significance included on differential diagnosis | 28 (21.7%) | 2 (4.7%) |
| Neutropenia associated with acute illness[1] | 42 (32.6%) | 15 (34.9%) |
| Outpatient evaluation | 102 (79.1%) | 28 (65.1%) |
| Isolated Neutropenia | 45 (34.9%) | 4 (9.3%) |
| **Hematologist concerns[2]:** | | |
| Anemia | 22 (17.1%) | 23 (53.5%) |
| Thrombocytopenia | 20 (15.5%) | 15 (34.9%) |
| Elevated cell count | 2 (1.6%) | 2 (4.7%) |
| Concern for blast cells | 0 (0%) | 5 (11.6%) |
| Hypogammaglobulinaemia | 1 (0.8%) | 2 (4.7%) |
| History of thrombosis | 2 (1.6%) | 0 (0%) |
| Other hematological problem | 4 (3.1%) | 7 (16.3%) |
| Fever | 28 (21.7%) | 8 (18.6%) |
| Immune dysfunction | 15 (11.6%) | 1 (2.3%) |
| “B” symptoms | 6 (4.7%) | 3 (7%) |
| Bone pain | 2 (1.6%) | 3 (7%) |

| | | |
|---|---|---|
| Lymphadenopathy | 2 (1.6%) | 1 (2.3%) |
| Other symptoms | 12 (9.3%) | 2 (4.7%) |
| Rheumatologic disease | 14 (10.9%) | 5 (11.6%) |
| Splenomegaly | 2 (1.6%) | 0 (0%) |
| Trisomy 21 | 2 (1.6%) | 0 (0%) |
| Other comorbidities | 9 (7%) | 1 (2.3%) |

Footnotes:

1. The neutropenia was initially observed as part of a work-up of an acute clinical presentation, typically suggestive of an infection, necessitating clinical attention.
2. Hematologist concerns explanations:
    a. “Anemia” or “thrombocytopenia”: indicated by the treating hematologist as additional hematological indications for performing the biopsy.
    b. "Concern for blast cells": Blast cells seen on peripheral smear.
    c. "Other hematological problem": Includes Alpha thalassemia; hemachromatosis, B12 deficiency, and bruising.
    d. "Fever": Patient had symptoms of recurrent or persistent fever(s).
    e. "Immune dysfunction": Patient history revealed pattern of persistent concerning infections.
    f. "B symptoms": Constellation of symptoms that included fevers, weight loss, night sweats.
    g. "Other symptoms": Includes Knee swelling; malar rash, emesis, diarrhea, renal failure, skin rash, epilepsy, periorbital cellulitis, congenital hypothyroidism, progressive dyspnea on exertion, shrunken thymus.
    h. "Rheumatologic disease": Crohn's disease, rheumatoid arthritis, felty syndrome, systemic lupus erythematous, Sjogren's syndrome, primary billiary cirrhosis, positive antihistone antibodies Goodpasture's disease, discoid lupus, positive ANA, mixed connective tissue, positive anti-DS DNA antibodies.

**Supplementary table 2: Population characteristics, stratified by whether the final hematological diagnosis was clinically-insignificant neutropenia or not.**

| Variable | Clinically-insignificant Neutropenia of (n=37) | Other diagnosis (n=135) | Univariate p-value by logistic regression |
|---|---|---|---|
| Black race | 25 (67.6%) | 17 (12.6%) | $9x10^{-10}$ |
| Males | 20 (54.1%) | 66 (48.9%) | 0.58 |
| Age (years) [Median (IQR)] | 30.3 (6.8 - 45.4) | 12.8 (2.0 - 45.1) | 0.2 |
| WBC count (x1000/mm3) [Median (IQR)] | 3.4 (2.9 - 4.4) | 3.0 (1.9 - 4.4) | 0.2 |
| ANC (x1000/mm3) [Median (IQR)] | 1.0 (0.7 - 1.3) | 0.5 (0.1 - 0.9) | 0.001 |
| Platelet count (x1000/mm3) [Median (IQR)] | 221 (197 - 264) | 210 (136 - 308) | 0.25 |
| Hemoglobin (g/dl) [Median (IQR)] | 12.6 (11.3 - 13.5) | 11.6 (9.9 - 13.1) | 0.009 |
| Outpatient evaluation | 36 (97.3%) | 94 (69.6%) | 0.008 |
| Isolated neutropenia | 20 (54.1%) | 29 (21.5%) | 0.0002 |
| Neutropenia of no clinical significance included on differential diagnosis | 18 (48.6%) | 12 (8.9%) | 0.0002 |
| Neutropenia associated with acute illness | 3 (8.1%) | 54 (40%) | 0.001 |
| **Hematologist concerns:** | | | |
| Anemia | 6 (16.2%) | 39 (28.9%) | 0.13 |
| Thrombocytopenia | 1 (2.7%) | 34 (25.2%) | 0.02 |
| Elevated cell count | 1 (2.7%) | 3 (2.2%) | 0.86 |
| Concern for blast cells | 0 (0%) | 5 (3.7%) | 0.99 |
| Hypogammaglobulinaemia | 1 (2.7%) | 2 (1.5%) | 0.62 |
| History of thrombosis | 2 (5.4%) | 0 (0%) | 0.99 |
| Other hematological problem | 2 (5.4%) | 9 (6.7%) | 0.78 |
| Fever | 1 (2.7%) | 35 (25.9%) | 0.01 |
| Immune dysfunction | 2 (5.4%) | 14 (10.4%) | 0.37 |
| “B” symptoms | 3 (8.1%) | 6 (4.4%) | 0.38 |
| Bone pain | 1 (2.7%) | 4 (3%) | 0.93 |
| Lymphadenopathy | 0 (0%) | 3 (2.2%) | 0.99 |

| | | | |
|---|---|---|---|
| Other symptoms | 0 (0%) | 14 (10.4%) | 0.99 |
| Rheumatologic disease | 0 (0%) | 19 (14.1%) | 0.99 |
| Other comorbidities | 2 (5.4%) | 8 (5.9%) | 0.91 |
| Splenomegaly | 0 (0%) | 2 (1.5%) | 0.99 |
| Trisomy 21 | 1 (2.7%) | 1 (0.7%) | 0.36 |
| Any Hematological comorbidity | 11 (29.7%) | 66 (48.9%) | 0.04 |
| Any symptom | 7 (18.9%) | 58 (43%) | 0.01 |
| Any comorbidities | 3 (8.1%) | 27 (20%) | 0.1 |

**Supplementary Table 3: Association between Black race and a diagnosis of clinically-insignificant neutropenia.** Shown are the results of a logistic regression models showing the 1) unadjusted association of Black race, and 2) the association adjusted for a propensity score for Black race. Variables contributing the propensity score were age, age-squared, ANC, ANC-squared, hemoglobin levels, platelet count, a clinical diagnosis of anemia, a clinical diagnosis of thrombocytopenia, presence of any hematological abnormality, presence of any symptom, presence of any other comorbidity, evaluation in inpatient setting, neutropenia associated with acute illness. Two participants (1 Black and 1 White) were excluded from these analyses because of extreme outlying propensity score. Inclusion of these 2 individuals gave a propensity-adjusted odds-ratio for Black race of 8.3 (95% CI: 3.3 - 21.2)

| Model | Covariate | Odds-ratio | 95% CI | p-value |
|---|---|---|---|---|
| Unadjusted | | | | |
| | Black race | 15.1 | (6.5 - 37.7) | $1.2\times10^{-9}$ |
| | | | | |
| Propensity score adjusted | | | | |
| | Black race | 7.9 | (3.1 - 21.1) | $2.2\times10^{-5}$ |
| | Propensity score | 375 | (23 - 8106) | $6.6\times10^{-5}$ |

**Distribution of propensity score values for Black and White participants.**

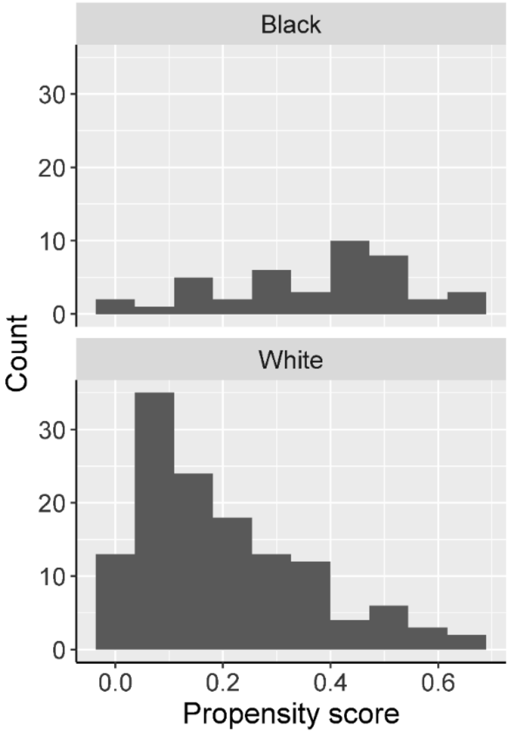

Supplement: Supplementary file 1 — Supplementary Material [file JCMM-26-3628-s001.pdf]
